# Effect of Breed on Transcriptional and Protein Expression of Lipogenic Enzymes in Tail and Subcutaneous Adipose Tissue from Two Grazing Breeds of Lambs

**DOI:** 10.3390/ani9020064

**Published:** 2019-02-15

**Authors:** María Gallardo, Luis Arias-Darraz, Juan Cárcamo

**Affiliations:** 1 Escuela de Medicina Veterinaria, Facultad de Ciencias, Universidad Mayor, Santiago PO Box 8580745, Chile; mugallar@gmail.com; 2 Instituto de Bioquímica y Microbiología, Facultad de Ciencias, Universidad Austral de Chile, Valdivia PO Box 567, Chile; l.arias.darraz@gmail.com

**Keywords:** breed, transcriptional expression, protein expression, tail fat, subcutaneous fat

## Abstract

**Simple Summary:**

An experiment to determine the effect of sheep breed on subcutaneous fatty acid composition was carried out at the Butalcura Research Station, Chiloé, Chile. To this end, two breeds of lambs were challenged to graze a typical, naturalized pasture of the Chiloé Archipelago, Chile, from 60 d to 120 d after birth. The animals were sacrificed to collect samples from subcutaneous fat (SCF) from the back, and tail fat (TF) to determine the effect of breed on transcriptional expression of lipogenic enzymes and fatty acid profile in these two fat depots. The results showed that although mRNA expression of enzymes was similar in both breeds, there were differences in certain protein levels in the SCF, partially related with the fatty acid profiles, thus affecting the selection of lamb breed either for human consumption or experimental purposes.

**Abstract:**

This experiment was carried out to determine the effect of breed on mRNA and protein expression levels of lipogenic enzymes acetyl-CoA carboxylase α (ACC), fatty acid synthase (FAS), stearoyl-CoA desaturase 1 (SCD1) plus sterol regulatory element binding transcription factor 1c (SREBP1c) in the subcutaneous fat (SCF) from the back of the animal, and tail fat (TF) of both Chilota and Suffolk Down lambs grazing Calafatal. Eight Chilota and six Suffolk Down 2-month-old male lambs were allocated to graze a “Calafatal”, a typical secondary succession of Chiloé Archipelago, Chile. After 62 d, lambs were slaughtered according to Chile’s meat industry standards. Fatty acid profile, RT-qPCR, and Western blot analyses from SCF and TF samples were performed. Although the mRNA expression levels of ACC, FAS, SCD1 and SREBP1c in SCF did not differ significantly between breeds (*p* > 0.05), a trend to higher mRNA expression of FAS and SREBP1c in TF from Chilota lambs was observed (*p* = 0.06). On the other hand, FAS levels in SCF were higher in Chilota than in Suffolk Down lambs (*p* < 0.02), although Suffolk Down showed higher fat contents and saturated fatty acid (SFA) proportions than Chilota lambs (*p* < 0.01). The FAS protein expression in TF was similar in both breeds (*p* > 0.05). Although the fat content was higher in Suffolk Down than in Chilota lambs (*p* < 0.01), the SFA proportions were similar in both breeds. Finally, it can be concluded that although mRNA expression of enzymes was similar in both breeds, there were differences in some protein levels in the SCF, partially related with the fatty acid profiles, thus affecting the selection of lamb breed either for human consumption or experimental purposes.

## 1. Introduction

Chilota, a sheep breed present only in Chiloé Archipelago, Chile, is the product of an intensive genetic differentiation and environmental adaptation process [1], acquiring some unique physical and functional characteristics which have allowed it to be recognized it as a new breed of sheep [2], and nowadays, it constitutes one of the last remnants of Iberian genetic traits without any selection.

After an adaptive process of this type, a high rusticity, not only of health, but also concerning feed intake [3], is expected. In Chiloé Archipelago, the main feed resource for domestic and wild animals is the Calafatal pasture (CP), a secondary succession dominated by shrubs which derives from the anthropic intervention of the native forest coupled to low sheep grazing intensities (2 o.e. ha^−1^ year. The ovine equivalent (o.e.) is a measure of the stocking rate corresponding to the energetic requirements of a sheep (55 Kg LW) rearing a 100-day-old suckling lamb [4].

It has been reported that the fatty acids present in the meat are influenced by the breed [5,6]. However, unlike feeding concentrates, a diet based on forage increases the *n*-3 polyunsaturated fatty acids (*n*-3 PUFA) proportions in the meat [5,7,8,9], thus improving food quality intended for human consumption [10]. Changes in the fatty acid profile, related to increased PUFA proportions, could be explained by changes in the expression levels of some genes associated with fatty acid metabolism [11,12,13]. In this regard, a tissue-specific response has been described in both muscle and adipose tissues [6].

To date, regarding the effect of breed on the transcriptional expression of genes in sheep, there are few studies comparing mRNA expression of lipogenic enzymes between breeds [14] or between species [15]. Our hypothesis is that the lamb breed should influence the transcriptional expression levels of lipogenic enzymes in the subcutaneous fat (SCF) from the back of the animal, and tail fat (TF), which is ultimately reflected on the fatty acid profiles. The objective of this study was to evaluate the effect of lamb breed on mRNA and protein expression levels of some key lipogenic enzymes, such as acetyl-CoA carboxylase α (ACC), fatty acid synthase (FAS), stearoyl-CoA desaturase 1 (SCD1), and sterol regulatory element binding transcription factor 1 (SREBP1c) and its final effect on the fatty acid profile in SCF and TF from Chilota and Suffolk Down lambs fed on Calafatal.

## 2. Materials and Methods

### 2.1. Animals and Sampling

The experiment was conducted at the Butalcura Research Station (Chiloé, Chile) from October to December 2011. The methodology used in this study was approved by the Committee for the Ethical Use of Animals in Experiments of the Universidad Austral de Chile. This study was part of the doctoral thesis of Maria Gallardo, which was approved by the Bioethics Committee of the Universidad Austral de Chile (N°09/2012). Fourteen 2-month-old lambs, i.e., eight Chilota, and six Suffolk Down, uncastrated males, no twin, similar body condition score (BCS) and their mothers, were randomly selected from a large free-grazing flock of Chilota and Suffolk Down sheep and subjected to grazing on CP. No significant differences in live weight (LW) or BCS between treatments, at the beginning (LW and BCS mean: 14.91 ± 0.79 kg and 2.74 ± 0.08, respectively) and at the end of the experiment (LW and BCS mean: 32.10 ± 2.22 kg and 3.19 ± 0.14, respectively), were found. After 62 d, the lambs were slaughtered in MAFRISUR slaughterhouse, according to Chile’s meat industry standards, using electric desensitization and fast bleeding by carotid arteries puncture.

Immediately after slaughtering, the animals were sampled, taking 1 g SCF from the left side of the carcass at a height between the 10th and 13th rib, and 1 g TF; the samples were kept in liquid nitrogen until analysis.

### 2.2. Lipid Extraction and Fatty Acid Analysis

Fatty acids were determined from SCF and TF samples. Total lipids were extracted in duplicate according to a modified procedure [16]; 1 g SCF and 1 g TF were extracted with methanol/dichloromethane/distilled water (2:1:0.8; *v*/*v*/*v*) by homogenization (Ultra Turrax, 3 × 15 s, 12,000 rpm) at room temperature, and methylation with methanolic NaOH solution. The fatty acid analysis of the fatty acid methyl esters (FAME) were performed using a capillary GC column (RT-2560, 100 m × 0.15 mm, 0.20 µm, Supelco, USA) installed in a Shimadzu gas chromatograph (GC 2010, Shimadzu, Japan) with a flame ionization detector and split injection. The initial oven temperature was 140 °C, and the temperature was increased to 240 °C at a rate of 3 °C min^−1^. Helium was used as the carrier gas at a flow rate of 1 mL min^−1^. The split ratio was 1:100, and the injector and detector were set at 250 °C and 280 °C, respectively. A reference standard FAME mixture C4-C24 (1000 µg/mL each component in *n*-hexane, analytical standard, 49453-U, Supelco, USA), C4-C24 unsaturated (wt. % varied, analytical standard, 18919-1AMP, Supelco, USA), linoleic acid, and conjugated linoleic methyl ester (05632, Sigma, USA) were used. All solvents and other chemicals used for GC were of HPLC grade.

### 2.3. qRT-PCR Analysis

Briefly, qRT-PCR analysis was accomplished using a Lightcycler Mx3005P (Agilent Technologies, California, CA, USA). RNA was extracted from 50 mg of SCF and TF tissue samples, analyzed for quantity/purity, and reverse transcribed with the M-MLV Reverse Transcriptase cDNA Synthesis Kit (Invitrogen, California, CA, USA). Subsequent qRT-PCR analysis was performed by subjecting reaction mixes of 5 μL Brilliant II SYBR^®^ Green Master Mix (Agilent Technologies, California, CA, USA), 0.5 μL forward/reverse (which were designed using Primer-BLAST and further analyzed by the Amplifx tool), primer solution (0.2 μmol/L), and 1 μL cDNA template (diluted 1:5) to a thermo cycling program for 10 s at 95 °C, 30 s at 60 °C, and 45 s at 70 °C (45 cycles). Specific oligonucleotides for ACC, FAS, SCD1, and SREBP1c genes were designed (Table 1) [15]. Relative mRNA expression was calculated with the comparative efficiency-corrected ΔΔCT method [17]. β-actin gene was stably expressed and served as a reference gene for gene expression normalization in these experiments. *p*-values were calculated for all comparisons.

### 2.4. Western Blot Analysis

In a few words, 200 mg of each SCF and TF tissue were added to 500 µL of RIPA buffer (50 mM Tris-HCl, pH 7.4; 150 mM NaCl; 1% Nonidet P-40; 1 mM EDTA; 1 mM EGTA), supplemented with 1× mixture of protease inhibitors and 10 mM of PMSF, (Winkler, USA), homogenized and frozen in liquid nitrogen and sonicated for 5–10 s. The tissue homogenate was centrifuged at 13,000 rpm for 30 min at 4 °C. The supernatant (~300 µL) was collected and stored at −20 °C. The protein fractions were quantitated using the Bicinchoninic Acid Assay (BCA). An aliquot containing proteins (25 µg/lane) was added to an equal volume of 2× loading buffer, heated at 95 °C for 5 min, and the proteins were separated by electrophoresis in a 6% SDS-PAGE for 30 min at 70 V, and 130 min at 90 V until the fall of the front. Following electrophoresis, the samples were transferred at 400 mA onto a PDVF (FAS, SCD1) or nitrocellulose membrane (ACC, SREBP1c, actin) for 1.5 h. Non-specific binding sites were blocked with 5% BIO-RAD Blotting-Grade Blocker in TBS- 0.05% Tween, for 1 h at room temperature. The polyclonal antibodies to ACC (H76, sc-30212, Santa Cruz Biotechnology, dilution 1:300), FAS (H-300, sc20140, Santa Cruz Biotechnology, dilution 1:100), SCD1 (H300, sc-30081, Santa Cruz Biotechnology, dilution 1:300), and SREBP1c (A-4, sc-365513, Santa Cruz Biotechnology, dilution 1:300) were diluted in a 1X PBS and 0.1% Tween 20 antibody dilution buffer containing 0.1% casein, 1x PBS, and 0.1% Tween 20. The nitrocellulose membrane was incubated with primary antibodies overnight at 8 °C. The membrane was washed with TBST. All membranes were incubated with the secondary antibody donkey anti-rabbit IgG-HRP (sc-2077, Santa Cruz Biotechnology), except SREBP1c which was incubated with anti-mouse antibody (610-1302, Rockland) diluted 1:5000 for 1 h at room temperature, followed by washing three times with TBST and one time with TBS. Western blot analyses were performed to pooled samples formed by triplicate extractions from each sample, being each lamb breed represented by 4 to 6 samples, and applied to all of the 12 or 14 protein samples from Chilota and Suffolk Down lambs in each gel. Antibodies were detected using a Westar Supernova chemiluminescent substrate (Cyanagen). Membranes were scanned with an Imager Genesys V1.2.8.0, and band intensities were densitometrically evaluated using ImageJ [18]. To exclude chemiluminescent protein signal intensity differences as factors influencing protein expression data, individual protein expression values were normalized to β-actin intensity (Thermo, PA5-16914, diluted 1:5000). The intensity variability of the β-actin band in each lane was due to the inhomogeneous performance by each of the sample extractions; however, by taking the precaution of avoiding the use of β-actin and target protein results from different extractions during the analyses, the internal proportion between β-actin and target protein was not affected. Since the size of SCD1 and β-actin is similar, a new (not stripped) membrane was employed, taking the precaution of using the same extraction for each analysis.

### 2.5. Statistical Analysis

To assess the breed effect on the fatty acid profile, mRNA and protein expression, the least square means were estimated using the general linear model procedures (GLM) of SAS statistical software (Version 9.1.3, SAS Institute Inc., 2006). T-test was performed to assess significant differences between means at *p*-value ≤ 0.05. The statistical model used was: y_ij_ = μ + t_i_ + e_ij_, where y_ij_ = observation ij; μ = the overall mean; t_i_ = the effect of breed_i_, and e_ij_ = random error; i = 1, 2; and j = 1, …, n. Relative gene expression was calculated with the comparative efficiency-corrected ΔΔCT method (REST^©^ 2009; Relative Expression Software Tool, Version V2.0.13); and *P*-values were estimated with the fold change data.

## 3. Results

### 3.1. Relative mRNA Expression

The relative mRNA expression levels of ACC, FAS, SCD1, and SREBP1c in SCF and TF from Chilota and Suffolk Down lambs were similar (*p* > 0.05) (Table 2). However, FAS and SREBP1c showed a trend (*p* = 0.06) to be upregulated in TF from Chilota in contrast to Suffolk Down lambs.

### 3.2. Protein Expression

**Figure 1:**
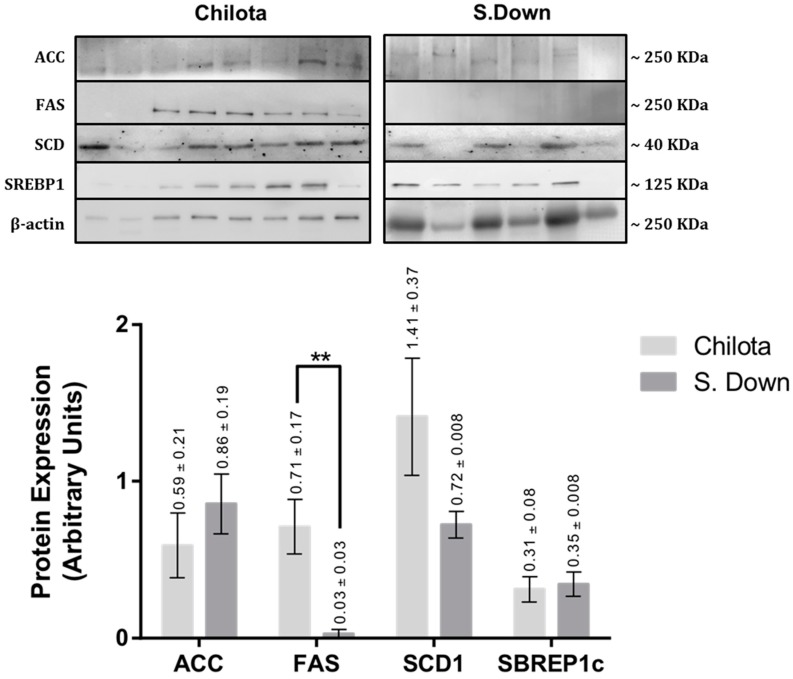
Protein expression levels of ACC, FAS, SCD1, and SREBP1c in subcutaneous fat from Chilota (*n* = 8) and Suffolk Down lambs (*n* = 6) grazing Calafatal pasture; analyzed by western blot. The LSM ± SEM values are shown above each bar of the graph. Normalized to β actin expression; ** *p* < 0.05.

**Figure 2:**
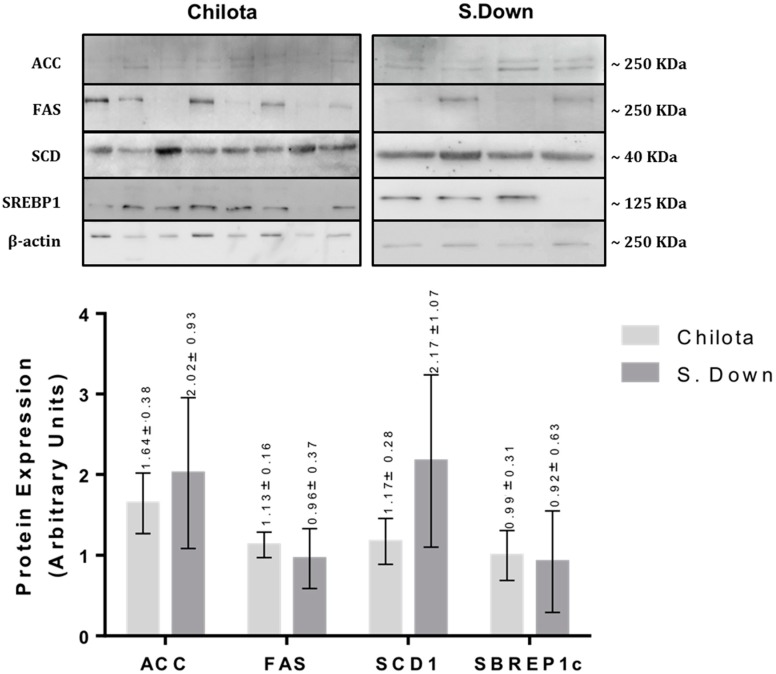
Protein expression levels of ACC, FAS, SCD1 and SREBP1c in tail fat from Chilota (*n* = 8) and Suffolk Down (*n* = 4) lambs grazing Calafatal pasture; analyzed by western blot. The LSM ± SEM values are shown above each bar of the graph. Normalized to β actin expression; *p* < 0.05.

The protein expression analyses of SCF samples (Figure 1) showed that the breed effect was only observed in FAS protein expression levels (0.71 ± 0.17 *vs.* 0.03 ± 0.03 AU, respectively; *p* < 0.02). The protein expression analyses of TF (Figure 2) did not show any significant difference between breeds (*p* > 0.05).

### 3.3. Fatty Acid Composition.

#### 3.3.1. Fatty Acid Composition in Subcutaneous Fat

Table 3 compares the fatty acid composition in SCF and TF of Suffolk Down lambs with the data reported for Chilota lambs grazing Calafatal. In Suffolk Down lambs, both SCF and TF showed different fat contents and fatty acid profiles. Regarding SCF, Suffolk Down lambs showed higher fat contents, sum of SFA (*p* < 0.01), and well as single fatty acids (FA) proportions 14:0, 16:0 and 18:0. On the other hand, Chilota lambs showed higher sum of PUFA (*p* < 0.03), sum of monounsaturated fatty acids (MUFA) (*p* < 0.01), and single FA concentrations 16:1 and 18:1 *cis*-9.

#### 3.3.2. Fatty Acid Composition in Tail Fat

In TF, the fat content was higher in Suffolk Down than in Chilota lambs (*p* < 0.01). The SFA proportions were similar in both breeds. The sum *n*-6 PUFA proportions were higher in Chilota than in Suffolk Down lambs, as well as the single FA 18:2 *n*-6 (*p* < 0.01). A trend to higher proportions of 20:4*n*-6 was observed in Chilota vs Suffolk Down lambs (*p* = 0.06). Although the sum *n*-3 PUFA proportions showed no significant differences between breeds, the Chilota lambs showed higher proportions of 18:3*n*-3 (*p* < 0.01), and also a trend to higher 20:3*n*-3 proportions was observed when compared to Suffolk Down lambs (*p* = 0.06).

## 4. Discussion

The mechanisms associated to fatty acid modification in ruminant adipose tissue have not been completely explained [19,20,21,22]. This situation is particularly true when looking at comparison between breeds [14,23,24]. In particular, there was some progress in the knowledge regarding the comparison between different species [15].

It is known that the changes in the fatty acid profile related to increases in the PUFA proportions could be explained by changes in the expression levels of some proteins related to fatty acid metabolism [11,12,13,25]. Previous studies have shown that *n*-3 PUFA supplementation decreases the gene expression of the mature form of SREBP1c, a transcription factor gene with a critical role in the transcriptional control of genes related to in vivo and in vitro fatty acid synthesis in the liver [26,27], and hence, reduces the expression of lipogenic genes, such as ACC and FAS [28,29], which play a role in the synthesis of triglycerides, a primary energy storage source and transport. During energy excess, ACC will convert acetyl CoA to malonyl-CoA, which is used by FAS to form palmitic acid, being able to be desaturated to palmitoleic acid (by SCD1) or elongated to stearic acid (by the long chain fatty acyl elongase). Stearic acid also can be desaturated to oleic acid (by SCD1) [30].

The mRNA expression levels measured showed no statistically significant differences at *p* < 0.05, although some differences were observed at *p* = 0.06, with 3.2- and 3.1-fold increases in FAS and SREBP1 levels of TF from Chilota lambs. However, these results do not reflect the levels of their corresponding proteins, confirming the information reported by Castro-Carrera et al. [31], who supplemented ewes with 25 g sunflower oil/kg diet and determined that the mRNA expression had a minimal contribution to the lipid metabolism in fat depots, and hence suggested that the response is mediated by other genes or post-transcriptional mechanisms. Regarding the effect of breed on the transcriptional level, Kashani et al. [32] working on 4 breeds of lambs supplemented with high and low spirulina proportions, reported no significant effects of breed or sex on the mRNA expression of FASN in the subcutaneous adipose tissue and *Longissimus* muscle.

Regarding the fatty acid composition, the statistically significant 23-fold increase in FAS levels of SCF from Chilota lambs, measured as Western Blot protein, would not be in agreement with a higher fat content and SFA proportion in this breed when compared to Suffolk Down lambs (*p* < 0.01). In fact, Chilota lambs showed a 40% of the stearic acid or 15% of the palmitic acid content present in SCF from Suffolk Down lambs (*p* < 0.01). However, Chilota lambs showed higher palmitoleic, oleic acid, CLAcis-9,trans-11 (57%), PUFA (12%), and MUFA (23%) proportions than Suffolk Down lambs, but this was not evidenced by a significant SCD1 protein increase (*p* > 0.12).

With respect to fatty acid composition in TF, an increase in linoleic and linolenic acid contents (14% and 20%, respectively) was observed in Chilota vs. Suffolk Down lambs. It is possible to argue that Chilota may have a better fixation of these two essential fatty acids obtained from the diet. The results obtained are reinforced by the total content of *n*-6 PUFA (Sum *n*-6 PUFA), showing a significant increase of 12% in TF of Chilota lambs, and a tendency to increase by 16% in the total content of *n*-3 PUFA (*p* = 0.09). In the case of SCF, no significant breed differences were observed for these parameters.

Regarding de novo synthesis, the higher sum SFA and single FA 14:0 16:0 and 18:0 found in SCF from Suffolk Down compared to Chilota lambs should be explained by an effect other than SREBP1c [33]. The transcriptional modulation performed by SRBP1 on its target genes is very complex and dependent on other transcriptional factors, modulator molecules and metabolic status. For example, the promoter regions of SCD1 genes have been characterized in various species such as human, mice, chicken, and bovine, noticing that numerous transcription factors can bind to the SCD1 promoter to perform a fine regulation of its expression [34]. The transcription factors include SREBP1c, LXR, PPAR-a, C/EBP-a, NF-1, NF-Y, AP-1, Sp1, TR and PGC1-a. Moreover, among modulator molecules and metabolic status, a high carbohydrate diet, insulin, peroxisome proliferators and cholesterol have been identified as positive effectors of SCD1 transcription whereas, triiodothyronine (T3), estrogen, PUFAs and leptin have been described as inhibitors. According to this, increased carbohydrate intake associated to diet, and depending upon carbohydrate composition, can strongly increase the endogenous fatty acid synthesis through increasing the expression of ACC, FAS and elongase Elovl6 [30,35], promoting the conversion of acetyl CoA into 16:0 and its elongation to 18:0 in SCF tissue of Suffolk Down lambs, which is in agreement with the higher fat content when compared to Chilota lambs. Ward et al. [23], working with Aberdeen Angus and Limousin crossbred steers reported a significant relationship between ACC and FAS expression and the SFA proportions, relating its amounts with the intramuscular fat content. It has been described that FAS protein expression would show specificity according to species and tissue [36,37].

It is known that ACC and FAS are key enzymes involved in de novo lipid synthesis, being SCD1 a critical enzyme related to desaturation processes [38] such as MUFA and conjugated linoleic acid (CLA) cis-9,rans-11 biosynthesis.

The analysis of protein expression showed higher FAS levels in SCF of Chilota vs Suffolk Down lambs; nonetheless, the expression levels of mRNAs were similar (*p* > 0.05). Differences between the expression levels of mRNAs and its respective proteins have been attributed to the mechanisms that contribute to the steady state level of the proteins, these include, RNA splicing, RNA transport, RNA stability, translation efficiency and protein stability [39]. The antibodies used were heterologous (ACC, FAS and SCD1 anti-rabbit, and SREBP1c anti-mouse), which could also be a source of discordance with the transcriptional expression. Thus, although not according to the transcriptional expression, the fatty acid profile was partially reflected by a higher expression of FAS in SCF of Chilota vs. Suffolk Down lambs. Xu et al. [40] reported special differences related to FAS protein expression between tail adipose and subcutaneous adipose tissues, and also between breeds, being somewhat consistent with their transcriptional expression, which did not happen in the present study.

On the other hand, in the present study we reported significant differences between breeds related to the sum PUFA in SCF (*p* < 0.03) and the sum *n*-6 PUFA in TF (*p* < 0.01). However, Maleki et al. [41] reported significant differences with respect to the sum PUFA and *n*-6 PUFA proportions in SCF but not in TF (*p* > 0.05) between breeds. Thus, the higher sum MUFA (*p* < 0.01) and sum PUFA (*p* < 0.03) found in SCF of Chilota vs. Suffolk Down lambs were not the result of a higher protein expression of SCD1 in this tissue [5,42]. However, the SCD1 protein expression levels could be influenced by breed and also by a tissue specific response of the sampled tissue [14], as in this case, by the SCF tissue. Dance et al. [6] studied the effect of genotype on the fatty acid composition and SCD1 expression in muscle and SCF tissue from 5 breeds of steers, and reported that the patterns which regulate SCD1 expression and CLA levels were tissue-specific.

## 5. Conclusions

Although the mRNA expression levels were similar in both lamb breeds, there were differences in the protein expression levels between them, which were partially related with the fatty acid profiles. This confirms that some breed-related variables are involved in determining the expression differences observed in the enzymes of the SCF and TF of these animals. This finding can be a useful parameter to determine the selection of a breed either for human consumption or experimental purposes; however, further research is necessary, including enzyme activity measurements, for a comprehensive clarification of the lipid metabolism in muscle and adipose tissues in lambs.

## Figures and Tables

**Table 1:** Primer specifications.

Gene	Forward Primer Sequence	Accession Number	Amplicon
	Reverse Primer Sequence		Length
ACC	F: ATGTGGCCTGGGTAGATCCT	NM_001009256.1	261 bp
	R: ACGTAACCAGGCTGATGGTG		
FAS	F: GGAAGGCGGGACTATATGGC	XM_004013447.1	278 bp
	R: CATGCTGTAGCCTACGAGGG		
SCD1	F: GGCGTTCCAGAATGACGTTT	NM_001009254.1	251 bp
	R: TGAAGCACAACAGCAGGACA		
SREBP1	F: GTCTACCACAAGCTGCACCAG	XM_004013336.1	216 bp
	R: GCTCAGGAAGAAGCGTGTCA		
β-Actin	F: TGAAGTGTGACGTGGACATCCGTA	NM_001009784.1	108 bp
	R: AGGTGATCTCCTTCTGCATCCTGT		

ACC, acetyl-CoA carboxylase alpha; FAS, fatty acid synthase; SCD1, stearoyl-CoA desaturase 1; SREBP1c, sterol regulatory element binding transcription factor 1c.

**Table 2:** Relative mRNA expression level differences (expressed as fold change, **FC**) in subcutaneous and tail fat samples from Chilota (*n* = 8) and Suffolk Down (*n* = 6) lambs grazing Calafatal pasture.

Breed	Subcutaneous Fat (SCF)		*p*	Tail Fat (TF)		*p*
	Chilota	Suffolk Down		Chilota	Suffolk Down	
Enzymes (FC)	LSM ^1^ ± SEM	LSM ^1^ ± SEM		LSM ^1^ ± SEM	LSM ^1^ ± SEM	
ACC	1.75 ± 0.78	1.50 ± 0.35	0.78	3.83 ± 1.71	0.64 ± 0.11	0.09
FAS	0.92 ± 0.29	1.49 ± 0.24	0.16	2.24 ± 0.70	0.70 ± 0.19	0.06
SCD1	1.07 ± 0.32	1.25 ± 0.17	0.63	1.66 ± 0.50	0.79 ± 0.08	0.12
SREBP1c	1.08 ± 0.34	1.50 ± 0.28	0.36	2.37 ± 0.77	0.76 ± 0.19	0.06

^1^ Normalization to β actin mRNA; *p* < 0.05.

**Table 3:** Fatty acid composition (%) of subcutaneous (from the back) and tail fats of Suffolk Down lambs compared with the reported data for Chilota lambs grazing Calafatal.

Breed	Subcutaneous Fat (SCF)			Tail Fat (TF)		
	Suffolk Down	Diff 1	*p*	Suffolk Down	Diff 2	*p*
Fatty Acids (%)	LSM ± SEM (*n* = 6)			LSM ± SEM (*n* = 6)		
Fat content (%)	40.65 ± 3.03	−2.82	0.01	67.57 ± 3.12	−4.49	0.01
14:0	6.77 ± 0.29	−1.38	<0.01	7.04 ± 0.32	−0.63	0.09
16:0	23.40 ± 0.49	−3.43	0.01	24.14 ± 0.54	−0.70	0.27
16:1	1.79 ± 0.18	+2.25	<0.01	2.17 ± 0.12	+0.03	0.86
18:0	20.64 ± 1.50	−8.15	0.01	20.90 ± 1.15	+0.76	0.63
18:1cis-9	38.39 ± 2.06	+9.80	0.01	36.41 ± 1.62	−0.06	0.98
18:2*n*-6	1.72 ± 0.11	−0.06	0.85	1.64 ± 0.05	+0.26	<0.01
18:3*n*-3	0.97 ± 0.04	+0.13	0.09	1.08 ± 0.04	+0.26	<0.01
CLA*cis*-9, *trans*-11	1.72 ± 0.39	+1.29	0.01	2.34 ± 0.15	−0.11	0.57
20:2*n*-6	0.03 ± 0.01	0.00	0.89	0.03 ± 0.01	0.00	0.35
20:3*n*-3	0.01 ± 0.00	0.00	0.92	0.01 ± 0.00	0.01	0.06
20:4*n*-6	0.25 ± 0.13	−0.15	0.21	0.14 ± 0.03	−0.06	0.06
20:5*n*-3	0.05 ± 0.01	+0.01	0.62	0.05 ± 0.01	−0.01	0.74
22:6*n*-3	0.09 ± 0.05	−0.04	0.39	0.05 ± 0.02	−0.01	0.20
Sum SFA ^†^	53.67 ± 2.09	−13.57	<0.01	54.94 ± 1.69	−0.35	0.88
Sum MUFA ^‡^	41.23 ± 2.13	+12.46	0.01	39.44 ± 1.64	−0.02	0.99
Sum PUFA ^§^	5.10 ± 0.39	+1.11	0.03	5.62 ± 0.23	+0.37	0.20
Sum *n*-6 PUFA ^¶^	2.17 ± 0.24	−0.30	0.36	1.98 ± 0.04	+0.26	0.01
Sum *n*-3 PUFA ^∥^	1.12 ± 0.09	+0.10	0.40	1.20 ± 0.05	+0.23	0.09
*n*-6/*n*-3PUFA ratio	2.07 ± 0.40	−0.52	0.25	1.65 ± 0.05	−0.09	0.30

^†^ Sum SFA (14:0; 15:0; 16:0, 17:0; 18:0; 20:0; 22:0; 23:0; 24:0); ^‡^ Sum MUFA (14:1; 15:1; 16:1; 17:1; 18:1*cis*-9; 18:1*trans*-9; 18:1*cis*-11; 20:1; 22:1; 24:1); ^§^ Sum PUFA (18:2trans; 18:2*n*-6; CLA*cis*-9, *trans*-11, 18:3*n*-3; 20:2*n*-6; 20:3*n*-6; 20:3*n*-3; 20:4*n*-6; 22:2*n*-6; 20:5*n*-3; 22:6*n*-3); ^¶^ sum *n*-6 PUFA (18:2*n*-6; 18:3*n*-6; 20:2*n*-6; 20:3*n*-6; 20:4*n*-6; 22:2*n*-6; ^∥^ sum *n*-3 PUFA: 18:3*n*-3; 20:3*n*-3; 20:5*n*-3; 22:6*n*-3; Diff 1: Difference 1: Chilota lambs minus Suffolk Down lambs; Diff 2: Difference 2: Chilota lambs minus Suffolk Down lambs (the fatty acid composition in SCF and TF from Chilota lambs grazing Calafatal has already been published (20)).
